# Relationship between Q-Tip Test and Urethral Hypermobility on Perineal Ultrasound

**DOI:** 10.3390/jcm12144863

**Published:** 2023-07-24

**Authors:** Cheng-Yu Long, Zi-Xi Loo, Ching-Hu Wu, Kun-Ling Lin, Chang-Lin Yeh, Chien-Wei Feng, Pei-Chi Wu

**Affiliations:** 1 Department of Obstetrics and Gynecology, Kaohsiung Medical University Hospital, Kaohsiung Medical University, Kaohsiung 80708, Taiwan; 2 Department of Obstetrics and Gynecology, Kaohsiung Municipal Siao-Gang Hospital, Kaohsiung Medical University, Kaohsiung 81267, Taiwan; 3 Graduate Institute of Medicine, College of Medicine, Kaohsiung Medical University, Kaohsiung 80708, Taiwan; 4 Department of Obstetrics and Gynecology, Kaohsiung Municipal Ta-Tung Hospital, Kaohsiung Medical University, Kaohsiung 80145, Taiwan; 5 Center for Cancer Research, Kaohsiung Medical University, Kaohsiung 807378, Taiwan; 6 Department of Obstetrics and Gynecology, National Taiwan University College of Medicine and Hospital, Taipei 100225, Taiwan

**Keywords:** Q-tip test, stress urinary incontinence, transperineal ultrasound, urethral hypermobility, urodynamic stress incontinence

## Abstract

Background: The aim of this study was to assess the correlation between the overall rest–stress distance measured by transperineal ultrasound (TPUS) and Q-tip test angle in women with urodynamic stress incontinence (USI), and determine a cut-off value of rest–stress distance for predicting urethral hypermobility (UH). Methods: Women with USI scheduled for mid-urethral sling surgery were retrospectively recruited. UH was defined as a Q-tip angle more than or equal to 30 degrees. Ultrasonic measurement of the overall rest–stress distance was defined as the linear distance of bladder-neck position change from resting status to maximal strain. Results: Among the 132 enrolled women, the Pearson correlation coefficient between the overall rest–stress distance in TPUS and Q-tip test angle was 0.9104 (95% CI, 0.8758–0.9357, *p* < 0.001). In receiver-operating-characteristic-curve analysis, a rest–stress distance of more than 13.3 mm was an optimal cut-off value to predict UH (sensitivity = 76.47%, specificity = 93.3%; area = 0.937, 95% confidence interval: 0.881–0.972). Conclusions: The overall rest–stress distance in TPUS correlated well with the Q-tip test angle, indicating that it can be an alternative method for the assessment of USI. A rest–stress distance of more than 13.3 mm was an optimal cut-off value to predict UH in women with USI.

## 1. Introduction

Stress urinary incontinence (SUI), defined as involuntary loss of urine on effort or physical exertion including sporting activities, or on sneezing or coughing [1], is one of the most common subjective complaints in the urogynecological clinic [2]. The number of patients with SUI is rising and this has attracted public attention [3]. SUI affects approximately 12.6% of women in developing countries. This rate is much higher than the rates for mixed urinary incontinence (MUI) (9.1%) and urgent urinary incontinence (UUI) (5.3%) [4]. In clinical terms, urinary leakage occurs if the intra-vesical pressure is higher than the urethral closure pressure. This is attributed to a decrease in urethral closure pressure caused by urethral dysfunction or increases in bladder pressure caused by detrusor dysfunction, or a combination of the two. Once urine leakage is confirmed during a urodynamic study (UDS) where there is increased abdominal pressure without detrusor contraction, the diagnosis of urodynamic stress incontinence (USI) is made [5]. Failure of the structural support of the urethra contributes to stress incontinence in women [6]. The unstable structure-related urethral hypermobility (UH) is associated with greater operative success after anti-incontinence surgery [7]. Therefore, the recognition of UH is an important part of female USI assessment, especially for preoperative counseling.

The Q-tip test was first described in 1971 as a simple method for UH assessment in an office setting [8]. With a cotton-tipped swab inserted in the urethra till bladder neck, the free end of the swab is rotated as the patient strains, correlating to the rotation of the urethrovesical junction. Measurement of the rotational degree is the Q-tip test result, and UH is identified if the straining angle is more than or equal to 30 degrees [7]. Although the Q-tip test is a useful tool, there is still concern regarding the discomfort associated with cotton-swab insertion. Additionally, this procedure can make the urethra rigid and potentially lead to non-homogeneous outcomes. Numerous patients felt uncomfortable during the performance of the test due to its invasiveness and clinicians may find the test difficult depending on their level of training and background. There is also a possible risk of infection in the urinary tract.

Transperineal ultrasound (TPUS) is a non-invasive technique for evaluating the pelvic anatomy with good reproducibility, and one of the approaches of pelvic floor ultrasound [6]. TPUS is used to assess the bladder-neck mobility and funneling of the internal urethral orifice. Most TPUS in women is performed in a supine lithotomy position, with the transducer sheathed in a protective covering and adequate gel used on the perineal area between the labia minora and labia majora. Due to the position, it is also called translabial ultrasound. The transducer should be located on the center of vaginal orifice, which is located posteriorly, and the urethral orifice, which is located anteriorly. During the past two decades, numerous measurements have been used to describe bladder-neck mobility, but the measurements were either indirect or complicated. Also, the correlations between the measurements and the Q-tip test were seldom addressed.

In this study, we evaluated UH with a simple measurement of linear rest–stress distance in TPUS for women with USI scheduled for mid-urethral sling surgery. We aimed to assess the correlation between the overall rest–stress distance and Q-tip test angle and determine a cut-off value of rest–stress distance for predicting UH.

## 2. Materials and Methods

Between January 2009 and January 2014, a total of 258 women with urodynamic stress incontinence (USI) scheduled for mid-urethral sling surgery in the urogynecological department of a tertiary referral center were recruited. A retrospective chart review was performed. This study received approval from the Institutional Review Board of Kaohsiung Medical University Hospital (ID:KMUHIRB-E(I)-20210275), and relevant guidelines and regulations were followed accordingly. We also registered this study on Clinicaltrials.org for better transparency (clinical trial identification number: NCT05205395). Informed consent was obtained from all participants before surgeries. The inclusion criteria was women with urodynamic stress incontinence (USI) scheduled for mid-urethral sling surgery. Women with more than or equal to stage 2 pelvic-organ prolapse (POP) defined by the POP-quantification system (POP-Q) [9] and receiving concomitant transvaginal mesh (TVM) surgery were excluded (*n* = 111). Women with incomplete ultrasound records were also excluded (*n* = 15). Ultimately, the analysis was conducted based on 132 available subjects with complete follow-up.

The baseline demographic data included age, parity, body mass index, past medical history, and previous surgical history. Informed consents for the examinations were obtained from all the patients. Every participant received urine analysis, a pelvic exam for POP, a Q-tip test, TPUS, and UDS. Urine leakage while increasing abdominal pressure, such as coughing and Valsalva, without simultaneous detrusor contraction during filling cystometry was deemed as USI. The Q-tip test was performed via a cotton swab with the patient in a 45-degree reclining position. After lubricating with 2% lidocaine jelly, the cotton swab was inserted with the tip over the bladder neck. The angle changes from rest to maximal straining were obtained three times, and the maximum angle change was recorded. In total, 102 women had angle changes of more than or equal to 30 degrees, while the remaining 30 women had angle changes of less than 30 degrees.

The TPUS was performed by two experienced urogynecologists (CY Long and KL Lin) according to KMUH-TPUS protocol. Voluson General Electric Sonography, expert 730 type (GE, Healthcare Ultrasound, Zipf, Austria) ultrasonography was used with a 3.5 MHZ curved linear-array transducer placed between the major labia and underneath the external urethral orifice. The measurement was done on the midsagittal plane to obtain an image of the pubic symphysis, urethra, and bladder in one screenshot. Ultrasonic measurement of the overall rest–stress distance was defined as the linear distance change in the bladder-neck position from resting status to maximal strain. The rest–stress distance was obtained three times, and the maximum distance was recorded.

Each of the two ultrasound operators (CY Long and KL Lin), blinded to each other’s data, measured every parameter twice, and the average of the two measurements was used for statistical analyses. Intra-observer reliability was evaluated using the measurements of the first investigator (CY Long), who performed two series of analyses (in both the out-patient clinic and before the operation) with an interval of 7–14 days between them, and was blinded to the previous analysis. Inter-observer reliability was assessed using the measurements of CY Long and KL Lin.

The primary outcome of this study was the correlation between the angle of the Q-tip test and the overall rest–stress distance of the bladder neck in TPUS. The secondary outcome was the optimal cut-off value of the rest–stress distance for prediction of UH.

IBM SPSS Statistical Software version 20.0 ed. was used for the statistical analyses. The Pearson correlation coefficient between the overall rest–stress distance in TPUS and Q-tip test angle was calculated. Receiver-operating-characteristic-curve analysis was applied to obtain an optimal cut-off value of rest–stress distance to predict UH. A *p*-value of less than 0.05 was considered statistically significant.

## 3. Results

A total of 132 women were included in the final analysis of this study. The demographic data are shown in Table 1. Among those stress-incontinent women, the mean age was 54.5 ± 11.3 years, and the mean parity was 2.5 ± 1.1. Ninety-three (70.5%) women were menopausal, and 19 (14.4%) women had received a hysterectomy before being recruited for this study.

**Figure 1:**
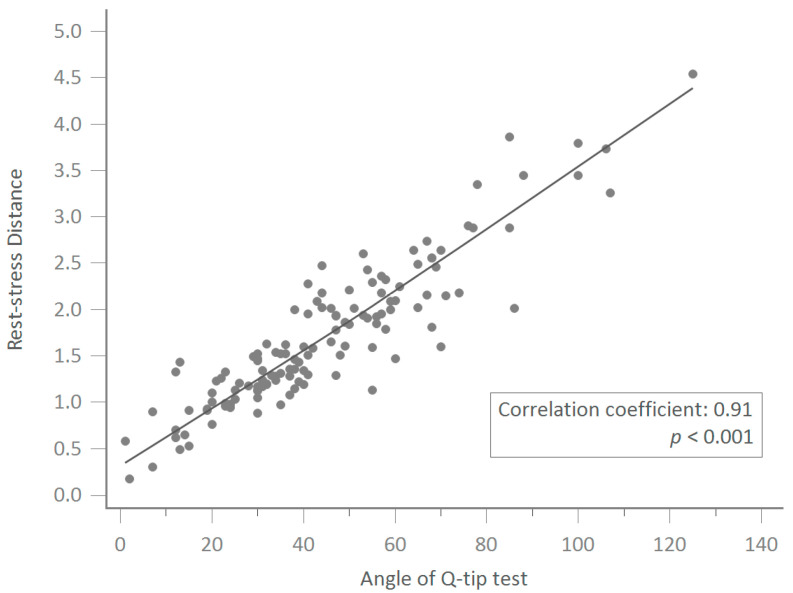
The Pearson correlation coefficient between the overall rest–stress distance in transperineal ultrasound and the Q-tip test angle was 0.9104 (95% CI, 0.8758–0.9357, *p* < 0.001).

The Pearson correlation coefficient between the overall rest–stress distance in TPUS and the Q-tip test angle was 0.9104 (95% CI, 0.8758–0.9357, *p* < 0.001) in the 132 women scheduled for MUS for their USI. (Figure 1).

**Figure 2:**
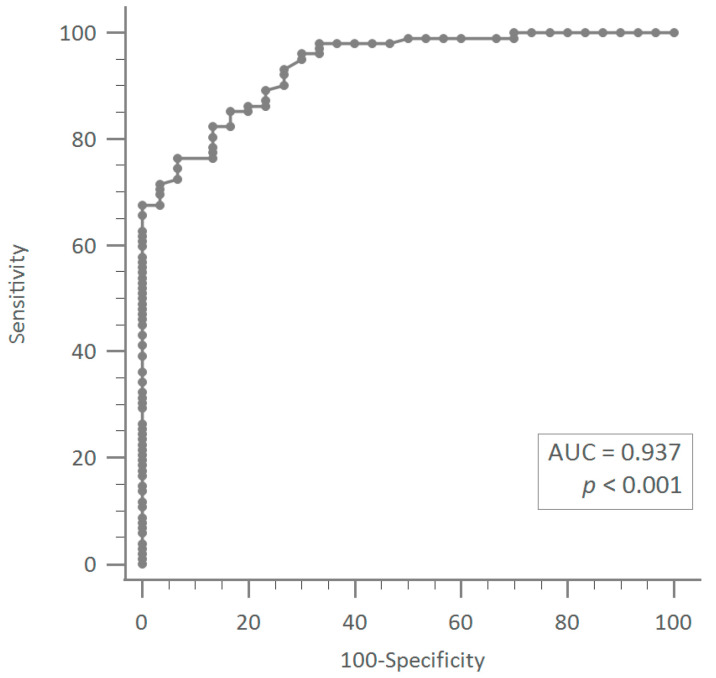
Receiver-operating-characteristic-curve analysis revealed that bladder-neck mobility of more than 13.3 mm was an optimal cut-off value to predict urethral hypermobility (sensitivity = 76.47%, specificity = 93.3%; area = 0.937, 95% CI: 0.881–0.972).

In receiver-operating-characteristic-curve analysis, bladder-neck mobility of more than 13.3 mm was an optimal cut-off value to predict UH (sensitivity = 76.47%, specificity = 93.3%; area = 0.937, 95% CI: 0.881–0.972) (Figure 2).

The positive and negative predictive values were 95.1% and 52%, respectively, using bladder-neck mobility of more than 13.3 mm as the cut-off value of the rest–stress distance in TPUS (Table 2). The positive likelihood ratio was 11.5, and the negative likelihood ratio was 0.25.

## 4. Discussion

Our study revealed that there was a good correlation between overall rest–stress distance in TPUS and Q-tip test angle. Therefore, the non-invasive measurement of rest–stress distance in TPUS may be applied as an alternative method to the Q-tip test in the assessment of USI and UH. Moreover, bladder-neck mobility with a rest–stress distance of more than 13.3 mm was an optimal cut-off value to predict UH in women with USI.

One of the anatomical causes of female SUI is identified as hypermobility of the bladder neck/urethra in response to increased abdominal pressure [10]. For more than 40 years, the Q-tip test has been applied in an office setting to quantify the urethral mobility of stress-incontinent women [8]. It is easy and reliable and, either with or without cystocele, the results do not alter the results significantly if it is performed correctly [11]. Greater than or equal to 30 degrees in Q-tip test results is more common in women with USI [12] and generally recognized as UH, although the definition has not yet been validated [13]. Although a useful tool and relatively less invasive compared to a cystogram and video urodynamic study [8], the Q-tip test is uncomfortable and may cause inflammation [7]. Thus, women with active urinary tract infection or inflammation were usually excluded from studies involving the Q-tip test [14]. Previous studies tried to access the Aa point in the POP-Q system as a proxy for the Q-tip test, but the Aa point and angle change in the Q-tip test did not have a good correlation [15], and UH cannot be predicted from Aa point alone [16]. Also, without a relatively clear anatomical reference during the insertion of the cotton swab, the tip of the swab may not be placed just at the bladder neck or the proximal urethra rather than inside the bladder, over mid-urethra, or distal urethra [11], leading to an inaccurate measurement. Besides, the results of the test vary with different bladder fullness statuses and patient positions [14]. Further less invasive and more comfortable assessment of USI and UH which correlates well with the Q-tip test should be investigated.

Ultrasound imaging has developed rapidly and been wildly applied in the urogynecological field in the evaluation of pelvic floor dysfunction, including urinary incontinence, since the 1980s [17]. The position and mobility can be accurately accessed by TPUS, providing real-time data in any situation, such as sitting, standing, coughing, and straining, to mimic daily activity. TPUS has been reported as a useful tool to evaluate female SUI and hypermobility of the bladder neck with a clear reference point of the pubic symphysis [18,19], although another ultrasonic approach was also reported [10]. TPUS is not routinely applied in predicting USI due to inadequate sensitivity, even though several parameters, including UH, were found significantly different between women with and without SUI [18,19]. Instead, the value of ultrasound is in the assessment of bladder-neck movement while increasing abdominal pressure in women with USI. By the same token, we previously reported the changes in bladder-neck mobility following hysterectomy, anti-incontinence surgery, and vaginal laser therapy using TPUS [20,21,22,23,24,25]. Our previous study showed assessed the efficacy on noninvasive erbium-doped yttrium aluminum garnet laser (Er:YAG laser) for female SUI. In total, 41 SUI patients were included in the study and results showed significant improvements in both urinary frequency and incontinence 6 months after Er:YAG laser treatment. Above all, measurements of bladder-neck mobility by TPUS significantly decreased (16.1 ± 6.4 mm to 10.5 ± 4.6 mm) after treatment which is consistent with all questionnaires including the Urinary Distress Inventory-6, the Incontinence Impact Questionnaire-7, The Overactive Bladder Symptom Score, and six questions on the inconvenience of POP [24]. This study showed that the bladder-neck mobility could directly reflect the condition of SUI patients.

The cotton swab in the Q-tip test is rigid, thus only the change of angle during straining can be calculated. On the contrary, the real-time data of ultrasound can clearly record the rotation angle of the proximal urethra, the curved change of the whole urethra, the bladder-neck position relative to the pubic symphysis, and the dynamic change after anti-incontinence surgery. Many studies have attempted to determine a cut-off value of UH in TPUS in women with USI; however, the settings of tests and measurements of reference point have varied [18,19,26,27]. Among all the ultrasonic parameters regarding hypermobility, the descent of bladder neck has the strongest association with USI [17,27]. Therefore, we tried to determine the cutoff value of rest–stress distance as a simple and accurate parameter for predicting UH and whether it correlates well with Q-tip angle. It was found that bladder-filling influences the mobility of the bladder neck [28]. Since standardized bladder volume allows reliable measurement, emptying the bladder and having the bladder checked before the measurement is the simplest method for standardization.

Unlike indirect measurement using accessory lines to determine the angles and distances [19], the measurement of bladder-neck mobility was simplified as linear rest–stress distance in this study. The result of this simple measurement correlated well with the Q-tip test, indicating that the non-invasive measurement of rest–stress distance in TPUS may be applied as an alternative method to the Q-tip test in the assessment of USI. Using the cut-off value of 13.3 mm in the rest–stress distance results in good specificity and positive predictive value for UH in women with USI. The high positive likelihood ratio also indicates the strong ability of rest–stress distance in identifying UH in this population. Using this simple measurement with good inter-observer and intra-observer reproducibility, medical caregivers can provide an important message in preoperative counseling of anti-incontinence surgery.

Some recent reports have also discussed the relationship between the Q-tip test and TPUS application in female SUI diagnosis. SUI can basically be separated into two categories—intrinsic sphincter deficiency (ISD) and/or urethral hypermobility, for which surgical treatment might be performed. Previous study suggested that there is not a correlation between abdominal leak-point pressure (ALPP) and urethral mobility degree and used the Q-tip test to confirm this. For this study, 221 SUI patients were recruited from 2014 to 2016. Using the International Consultation on Incontinence Questionnaire Short Form (ICIQ-SF), the Q-tip test, and invasive urodynamics, 65.3% were assessed as moderate and 6.8% as severe. Results demonstrated a 61.75%, 51.61%, and 70.6% agreement between ALPP and urethral mobility (UM), ALPP and composite A (equal to a Q-tip test < 30° AND ICIQ-SF ≥ 10 points), and ALPP and composite variable B (equal to low urethral mobility, AND/OR hypoestrogenism, AND/OR history of radiotherapy, AND/OR previous pelvic surgery), respectively. The authors found that neither the degree of UM, nor the composite variables, correlated or agreed with urethral function tests in UDS, which suggested that the ALPP cannot be predicted using the Q-tip test or the ICIQ-SF for classifying patients with SUI [29]. However, Ohter study in 2022 intended to evaluate the use of transperineal ultrasonography while diagnosing SUI by comparing the urethral angle (α), posterior urethrovesical angle (β), and bladder-neck descent (BND) during rest and Valsalva maneuver in continent women and women with SUI. They recruited 100 women in the study and separated them into SUI and non-SUI groups. Their data showed that both α and β angles were significantly higher in women with SUI during the Valsalva maneuver compared to the non-SUI group. Besides, the difference between rest and Valsalva of α and β angles (Rβ, Rα) was also significantly higher in women with SUI. Their results showed that the cut-off point for Rα in the diagnosis of SUI was 16° and the Q-tip test angle also demonstrated a positive correlation to Rα value. They also evaluated the pain with the Visual Analogue Scale (VAS) score during both examinations. The VAS scores for the Q-tip test were significantly higher than for TPUS. The BND data (r:0.847 p:0.000) also showed that the SUI group had a higher value and the cut-off point determined for BND in the diagnosis of SUI was >11 mm (90% sensitivity, 98% specificity) [30]. Our data also showed a similar trend (r:0.91 p:0.000) in BND. The cut-off value to predict UH (sensitivity = 76.47%, specificity = 93.3%; area = 0.937, 95% confidence interval: 0.881–0.972) in our data was a rest–stress distance of more than 13.3 mm, which gave a 7% higher specificity than the result in the previous investigation.

Our study revealed that it is practical and reliable to evaluate UH by the overall rest–stress distance in TPUS with a cut-off value of more than 13.3 mm. This study provides new and useful information for pretreatment counseling.

Since the more than or equal to 30 degrees in Q-tip angle change was just a generally applied but valid value, this may have some impacts on our results. Further study regarding postoperative change and anti-incontinence effect may help to clarify the clinical relevance of the ultrasonic findings of UH. As this is a new cut-off value of UH, validation studies are also necessary. Since the population in this study was mainly stress-incontinent women with little or no POP, whether advanced POP affects the results remains to be explored.

The retrospective design of this study was the major limitation. However, the large sample size may compensate for this weakness. The diagnostic accuracy in ultrasonic examination depends largely on the operator. We conducted the protocol with the same operators, equipment, patient position, and bladder filling volume, and this setting would provide more reliable data.

## 5. Conclusions

The overall rest–stress distance in TPUS correlates well with the Q-tip test angle, indicating that it appears to be an alternative method to the Q-tip test to assess women with USI. The rest–stress distance of the bladder neck of more than 13.3 mm was an optimal cut-off value to predict UH in women with USI.

## Figures and Tables

**Table 1:** Demographic characteristics of the women scheduled for mid-urethral sling surgery for their stress urinary incontinence (*n* = 132).

Parameters	Mean ± SD *n* (%)
Age (years)	54.5 ± 11.3
Parity	2.5 ± 1.1
BMI (kg/m^2^)	25.5 ± 4.6
Menopause	93 (70.5)
History of hysterectomy	19 (14.4)
Diabetes mellitus	8 (6.1)
Hypertension	24 (18.2)
Current smoker	2 (1.5)

**Table 2:** Sensitivity, specificity, positive predictive value, negative predictive value, and positive and negative likelihood ratios for diagnosis of urethral hypermobility using transperineal ultrasound with cutoff value of 13.3 mm.

	Bladder-Neck Mobility > 13.3 mm
Sensitivity	76.5%
Specificity	93.3%
Positive predictive value	95.1%
Negative predictive value	52.0%
Positive likelihood ratio	11.5
Negative likelihood ratio	0.25

## Data Availability

The authors confirm that neither the manuscript nor any parts of its content are currently under consideration for, or published in, another journal even in other languages.

## References

[1] Haylen B.T., Maher C.F., Barber M.D., Camargo S., Dandolu V., Digesu A., Goldman H.B., Huser M., Milani A.L., Moran P.A. (2016). An International Urogynecological Association (IUGA)/International Continence Society (ICS) joint report on the terminology for female pelvic organ prolapse (POP). Int. Urogynecology J..

[2] Reynolds W.S., Dmochowski R.R., Penson D.F. (2011). Epidemiology of stress urinary incontinence in women. Curr. Urol. Rep..

[3] Zhang R.Q., Xia M.C., Cui F., Chen J.W., Bian X.D., Xie H.J., Shuang W.B. (2021). Epidemiological survey of adult female stress urinary incontinence. BMC Women’s Health.

[4] Minassian V.A., Hagan K.A., Erekson E., Austin A.M., Carmichael D., Bynum J.P.W., Grodstein F. (2020). The natural history of urinary incontinence subtypes in the Nurses’ Health Studies. Am. J. Obstet. Gynecol..

[5] Nygaard I.E., Heit M. (2004). Stress Urinary Incontinence. Obstet. Gynecol..

[6] DeLancey J.O. (1994). Structural support of the urethra as it relates to stress urinary incontinence: The hammock hypothesis. Am. J. Obstet. Gynecol..

[7] Robinson B.L., Geller E.J., Parnell B.A., Crane A.K., Jannelli M.L., Wells E.C., Connolly A., Matthews C.A. (2012). Diagnostic accuracy of visual urethral mobility exam versus Q-Tip test: A randomized crossover trial. Am. J. Obstet. Gynecol..

[8] Bergman A., McCarthy T.A., Ballard C.A., Yanai J. (1987). Role of the Q-tip test in evaluating stress urinary incontinence. J. Reprod. Med..

[9] Bump R.C., Mattiasson A., Bø K., Brubaker L.P., DeLancey J.O., Klarskov P., Shull B.L., Smith A.R. (1996). The standardization of terminology of female pelvic organ prolapse and pelvic floor dysfunction. Am. J. Obstet. Gynecol..

[10] Johnson J.D., Lamensdorf H., Hollander I.N., Thurman A.E. (1992). Use of transvaginal endosonography in the evaluation of women with stress urinary incontinence. J. Urol..

[11] Karram M.M., Bhatia N.N. (1988). The Q-tip test: Standardization of the technique and its interpretation in women with urinary incontinence. Obstet. Gynecol..

[12] Walters M.D., Shields L.E. (1988). The diagnostic value of history, physical examination, and the Q-tip cotton swab test in women with urinary incontinence. Am. J. Obstet. Gynecol..

[13] Smith P.P., van Leijsen S.A., Heesakkers J.P., Abrams P., Smith A.R. (2012). Can we, and do we need to, define bladder neck hypermobility and intrinsic sphincteric deficiency? ICI-RS 2011. Neurourol. Urodyn..

[14] Yun J.H., Kim J.H., Park S., Lee C. (2015). Changes in the Q-tip angle in relation to the patient position and bladder filling. BMC Urol..

[15] Zyczynski H.M., Lloyd L.K., Kenton K., Menefee S., Boreham M., Stoddard A.M. (2007). Correlation of Q-tip values and point Aa in stress-incontinent women. Obstet. Gynecol..

[16] Mattison M.E., Simsiman A.J., Menefee S.A. (2006). Can urethral mobility be assessed using the pelvic organ prolapse quantification system? An analysis of the correlation between point Aa and Q-tip angle in varying stages of prolapse. Urology.

[17] Dietz H.P. (2004). Ultrasound imaging of the pelvic floor. Part I: Two-dimensional aspects. Ultrasound Obstet. Gynecol. Off. J. Int. Soc. Ultrasound Obstet. Gynecol..

[18] Pregazzi R., Sartore A., Bortoli P., Grimaldi E., Troiano L., Guaschino S. (2002). Perineal ultrasound evaluation of urethral angle and bladder neck mobility in women with stress urinary incontinence. BJOG Int. J. Obstet. Gynaecol..

[19] Chen G.D., Su T.H., Lin L.Y. (1997). Applicability of perineal sonography in anatomical evaluation of bladder neck in women with and without genuine stress incontinence. J. Clin. Ultrasound JCU.

[20] Long C.-Y., Hsu S.-C., Wu T.-P., Fu J.-C., Hsu Y.-S., Su J.-H. (2003). Effect of laparoscopic hysterectomy on bladder neck and urinary symptoms. ANZJOG.

[21] Long C.Y., Hsu S.C., Chang Y., Chen Y.C., Su J.H., Tsai E.M. (2004). The clinical and urodynamic effects of the tension free bladder neck sling procedure. Int. Urogynecology J. Pelvic Floor Dysfunct..

[22] Long C.Y., Liu C.M., Wu T.P., Hsu S.C., Chang Y., Tsai E.M. (2005). A randomized comparison of vesicourethral function after laparoscopic hysterectomy with and without vaginal cuff suspension. J. Minim. Invasive Gynecol..

[23] Lin K.L., Juan Y.S., Lo T.S., Liu C.M., Tsai E.M., Long C.Y. (2012). Three-dimensional ultrasonographic assessment of compression effect on urethra following tension-free vaginal tape and transobturator tape procedures. Ultrasound Obstet. Gynecol. Off. J. Int. Soc. Ultrasound Obstet. Gynecol..

[24] Lin K.-L., Chou S.-H., Long C.-Y. (2019). Effect of Er:YAG Laser for Women with Stress Urinary Incontinence. BioMed Res. Int..

[25] Lin K.L., Juan Y.S., Chou S.H., Long C.Y. (2019). Ultrasonographic Assessment with Three-Dimensional Mode of the Urethral Compression Effect following Sling Surgery with and without Mesh Surgery. BioMed Res. Int..

[26] Alper T., Cetinkaya M., Okutgen S., Kökçü A., Malatyalioğlu E. (2001). Evaluation of urethrovesical angle by ultrasound in women with and without urinary stress incontinence. Int. Urogynecology J. Pelvic Floor Dysfunct..

[27] Dietz H.P., Clarke B., Herbison P. (2002). Bladder neck mobility and urethral closure pressure as predictors of genuine stress incontinence. Int. Urogynecology J. Pelvic Floor Dysfunct..

[28] Dietz H.P., Wilson P.D. (1999). The influence of bladder volume on the position and mobility of the urethrovesical junction. Int. Urogynecology J. Pelvic Floor Dysfunct..

[29] Robledo D., Zuluaga L., Bravo-Balado A., Domínguez C., Trujillo C.G., Caicedo J.I., Rondón M., Azuero J., Plata M. (2020). Present value of the Urethral mobility test as a tool to assess Stress urinary incontinence due to Intrinsic sphincteric deficiency. Sci. Rep..

[30] Turkoglu A., Coskun A.D.E., Arinkan S.A., Vural F. (2022). The role of transperineal ultrasound in the evaluation of stress urinary incontinence cases. Int. Braz J Urol Off. J. Braz. Soc. Urol..

